# Interactions between the Nucleoprotein and the Phosphoprotein of Pneumoviruses: Structural Insight for Rational Design of Antivirals

**DOI:** 10.3390/v13122449

**Published:** 2021-12-06

**Authors:** Hortense Decool, Lorène Gonnin, Irina Gutsche, Christina Sizun, Jean-François Eléouët, Marie Galloux

**Affiliations:** 1 IM, UVSQ, INRAE, Université Paris-Saclay, 78350 Jouy-en-Josas, France; hortense.decool@inrae.fr (H.D.); lorene.gonnin@inrae.fr (L.G.); 2 IBS, CEA, CNRS, University of Grenoble Alpes, 38044 Grenoble, France; irina.gutsche@ibs.fr; 3 Institut de Chimie des Substances Naturelles, CNRS, Université Paris-Saclay, 91190 Gif-sur-Yvette, France; christina.sizun@cnrs.fr

**Keywords:** pneumoviruses, RSV, HMPV, nucleoprotein, phosphoprotein, protein-protein interaction, structure, antivirals, nucleocapsid

## Abstract

Pneumoviruses include pathogenic human and animal viruses, the most known and studied being the human respiratory syncytial virus (hRSV) and the metapneumovirus (hMPV), which are the major cause of severe acute respiratory tract illness in young children worldwide, and main pathogens infecting elderly and immune-compromised people. The transcription and replication of these viruses take place in specific cytoplasmic inclusions called inclusion bodies (IBs). These activities depend on viral polymerase L, associated with its cofactor phosphoprotein P, for the recognition of the viral RNA genome encapsidated by the nucleoprotein N, forming the nucleocapsid (NC). The polymerase activities rely on diverse transient protein-protein interactions orchestrated by P playing the hub role. Among these interactions, P interacts with the NC to recruit L to the genome. The P protein also plays the role of chaperone to maintain the neosynthesized N monomeric and RNA-free (called N^0^) before specific encapsidation of the viral genome and antigenome. This review aims at giving an overview of recent structural information obtained for hRSV and hMPV P, N, and more specifically for P-NC and N^0^-P complexes that pave the way for the rational design of new antivirals against those viruses.

## 1. The *Pneumoviridae* Family

Pneumoviruses belong to the *Mononegavirales* order that includes many pathogenic human or animal viruses in 11 families, such as respiratory syncytial virus (RSV), metapneumovirus (MPV), Measles, Mumps, Rabies, Nipah, Ebola, and Vesicular stomatitis viruses (VSV) [1]. *Mononegavirales* have a non-segmented negative-sense RNA genome ranging from 13.2 to 15.3 kb. They form a large group exhibiting common genome organization and sharing similar replication mechanisms. Recently, the former paramyxoviral subfamily *Pneumovirinae* was elevated to family status *Pneumoviridae* [2]. This “new” family is composed of the two genera, *Metapneumovirus* and *Orthopneumovirus* (Table 1) [3].

The *Metapneumovirus* genus includes human metapneumovirus (hMPV) and avian metapneumovirus (aMPV). The *Orthopneumovirus* genus groups human respiratory syncytial virus (hRSV), bovine respiratory syncytial virus (bRSV), and pneumonia virus of mice (PVM). Although unclassified by the International Committee on Taxonomy of Viruses (ICTV), this taxon also includes ovine respiratory syncytial virus (ORV) and canine pneumovirus (CPV). More recently, an eighth pneumovirus was identified by metagenomic sequencing of pooled nasal swabs in feral swine in the USA [4]. This newly identified *Orthopneumovirus* shows 93% and 91% protein identities with PVM and CPV, respectively, and was named swine orthopneumovirus (SOV). Amino acid sequence identities between nucleoproteins of SOV and other pneumoviruses are 59.8% for hRSV, 60% for bRSV, 45.7% for hMPV, and 43.3% for aMPV, respectively, indicating that the PVM/SOV group is distinct from *Meta-* and *Ortho-pneumoviruses* and could constitute a third genus.

## 2. Impact of Infections by *Pneumoviridae*

Viruses belonging to the *Pneumoviridae* family cause severe respiratory diseases in humans and animals. Among them, hRSV and hMPV are the main cause of bronchiolitis and pneumonia in young children (<5 years) [5,6,7,8]. hRSV infects nearly 100% of children in the first three years of life and is one of the principal causes of child hospitalizations. Worldwide, hRSV is estimated to be responsible for ~33 million acute lower respiratory infections (ALRI), resulting in more than 3.2 million ALRI-related hospitalizations and 118,200 deaths in children under 5 years [9]. In a recent systemic multisite study, hRSV was shown to be the first etiological agent responsible for severe pneumonia (more than 30%) in hospitalized children in Asia and Africa [10]. It is noteworthy that hRSV is also a frequent cause of otitis in infants [11] and that children who suffer from severe hRSV infection are at risk of developing further respiratory complications such as asthma [12].

After hRSV, hMPV is considered the second most common cause of ALRI in young children [7,8,13]. Isolated in 2001 in the Netherlands [14], it is thought to have derived from avian metapneumovirus (aMPV) subgroup C, 200 years ago [15]. The peak age of hospitalization for infants infected by hMPV occurs between 6–12 months, slightly later than the peak of hRSV, which is around 2–3 months. The clinical features and severity of hMPV are similar to those of hRSV. Furthermore, hRSV and hMPV are now recognized as being responsible for significant morbidity and mortality in elderly and immunocompromised persons, such as bone marrow transplant patients (with comparable disease burden to influenza) [16,17,18,19,20]. These viruses are seasonal, the peak of infection typically extending from early fall to early spring. In 2020, the emergence of coronavirus disease (COVID-19) triggered the large-scale implementation of non-pharmaceutical interventions such as confinement, mask-wearing, and extensive handwashing [21]. These preventive public health measures have had an impact on the circulation of diverse pathogens, specifically hRSV, as evidenced by the interseasonal epidemics of hRSV in several countries of the southern hemisphere and late epidemics of hRSV in the USA, Japan, and several European countries [22,23,24,25]. For example, the 2020–2021 bronchiolitis epidemic in mainland France lasted 15 weeks, comparable to the previous season, but with a delayed peak, 13 weeks later than that of the previous season, with a much lower amplitude. The proportion of hospitalizations for bronchiolitis has been comparable to that of recent seasons, but notable features of the 2020/21 season were a decrease in the proportion of cases over 65 years of age and an increase in the proportion of cases in children over 3 months and up to 5 years. In addition to the resurgence of hRSV and hMPV infections since March 2021, data indicate more severe illness in younger infants, possibly because of reduced immunity due to lack of exposure to these viruses in the previous season.

Finally, the *Pneumoviridae* family is also an important threat for livestock farming and has a strong economic impact, bRSV, and aMPV causing severe respiratory diseases in calves and poultry, respectively [26,27]. These infections are responsible for important animals‘ morbidity, leading to high mortality rates, mainly due to opportunistic infections by other viruses or bacteria [28,29]. To limit this, the current care consists of antibiotic administration during epidemics, which represents an indirect risk for animal and human health due to the emergence of resistant bacteria. In addition, the discovery of SOV in the USA suggests that yet unknown pneumoviruses could be responsible for respiratory diseases in other animal species. A recent study suggested a high prevalence of this virus in France [30]. However, further studies are required to determine whether this virus is pathogenic for pigs.

## 3. Treatments against Pneumoviruses

No vaccine is available against hRSV and hMPV. Although several vaccines against bRSV and aMPV are commercialized, their efficacy remains limited [27,31,32,33,34]. In this context, the development of antiviral drugs with a wide spectrum represents an alternative to human vaccination. This is especially true because vaccine development against hRSV and hMPV is hampered by the fact that these viruses mostly infect infants who have an immature immune system. Furthermore, as hRSV is 80% fatal to immunocompromised and transplanted patients and a significant cause of death in the elderly, the elaboration of antiviral strategies is a recognized necessity. So far, no specific inhibitors are commercially available against these viruses, ribavirin being used only exceptionally because of its toxicity and poor efficiency. A humanized monoclonal antibody directed against the surface fusion F glycoprotein (palivizumab Synagis^®^) is also available as a preventive treatment, but its efficiency is limited (≈50%), and its high cost restricts its use to high-risk infants [35]. Approximately 6000 children are treated with Synagis each year in France, with a cost of EUR 8000 per child (five injections). There is, therefore, a need for new and cheaper treatments, which implies the critical necessity to better understand the molecular mechanisms of virus replication. To date, most developed antiviral strategies aim at targeting the F protein to impair virus entry [36,37,38,39]. The viral polymerase L that is responsible for the enzymatic activities required for viral replication and transcription is the second main target of interest [40,41]. Among the developed compounds, the two fusion inhibitors, GS-5806 and JNJ-53718678, and the polymerase inhibitor ALS-008176, have been tested in humans [38,40,42]. However, the results of phase 2b trials of GS-5806 were disappointing [43,44], and clinical trials of ALS-008176 have recently been halted. The emergence of escape mutants upon treatment represents the main restriction and highlights the necessity to identify new targets and to associate different compounds. The functioning of the viral polymerase depends on different highly conserved transient protein-protein interactions (PPIs) that have no counterparts in cells. These viral PPIs being transient and of low affinity, molecules of high affinity that could compete with them may represent a new class of inhibitors. Furthermore, these interactions are now structurally well-characterized, allowing the rational structure-based design of antivirals.

## 4. Virions and Viral Cycle

**Figure 1:**
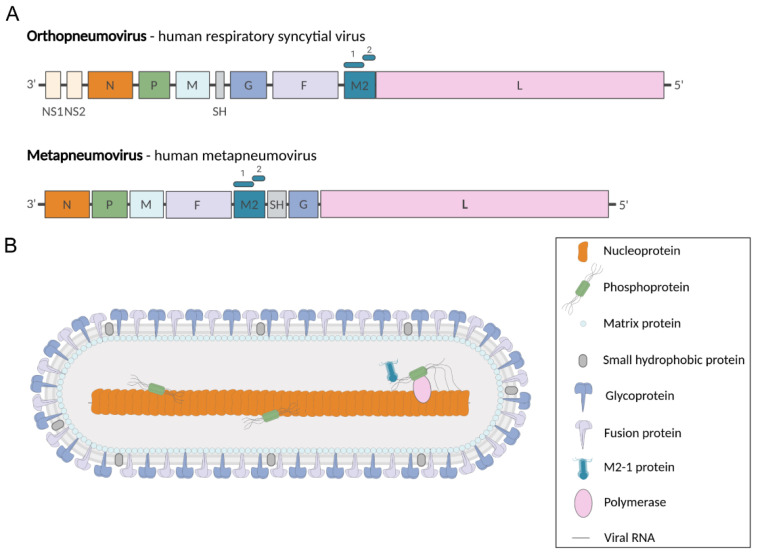
*Pneumoviridae* genomes and virion. (**A**) Genome organization of representative members of the *Pneumoviridae* family. Genomic RNAs are presented in sense (coding) orientation (3′-to-5′), with each box representing a gene encoding a separate mRNA drawn approximately to scale. The M2 gene encodes M1-2 and M2-2 proteins (represented by rectangles above M2 gene). (**B**) Representation of pneumovirus viral particle showing the structural proteins. Created with BioRender.com.

Pneumoviruses are enveloped viruses, the virions having pleomorphic but mostly filamentous shapes [45,46,47]. Their genomes contain 8 to 10 genes that encode 9 and 11 proteins in the case of MPV and RSV, respectively (Figure 1A). The two non-structural proteins NS1 and NS2 of RSV, which are involved in the control of antiviral pathways during infection [48,49], have no counterparts in MPV. The virions present three transmembrane proteins: the glycoprotein (G) involved in virion attachment to the cell surface, the fusion (F) protein responsible for receptor binding and fusion between viral and cellular membranes, and the small hydrophobic protein (SH), a viroporin whose immunomodulatory role still remains unclear [50,51,52] (Figure 1B). The inner side of the viral membrane is lined by the matrix (M) protein. The viral particles contain the genomic RNA encapsidated by the N protein, forming the nucleocapsid (NC), which is associated with the P-L-M2-1 proteins.

**Figure 2:**
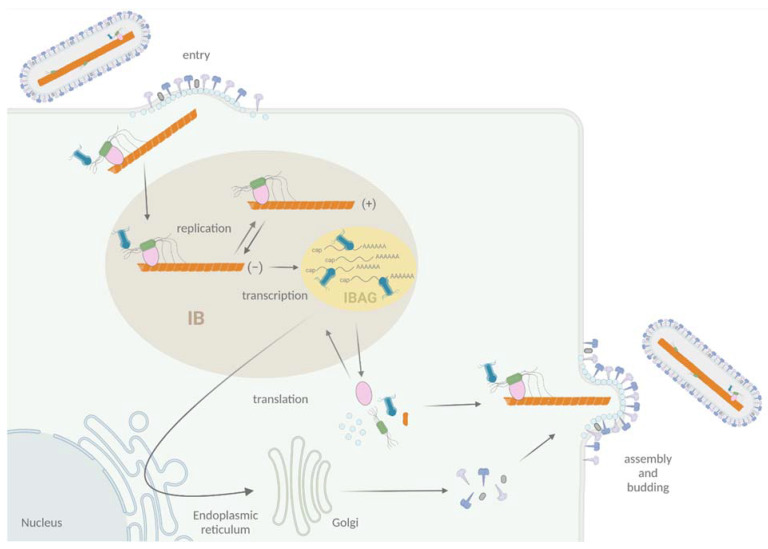
Schematic representation of the viral cycle of Pneumoviruses. Virion attachment to the cell is mediated by F and G proteins. The F protein is responsible for the fusion of viral and cell membranes, leading to the delivery in the cytoplasm of the NC complexed with L, P, and M2-1 proteins. Transcription and replication occur in membrane-less organelles called cytoplasmic inclusions bodies (IBs, light brown). Within IBs, M2-1 and viral mRNAs accumulate into sub-structures called inclusion body-associated granules (IBAGs, yellow). After viral protein production and genome replication, assembly and budding of new viral particles take place at the plasma membrane. Adapted from “Replication Cycle”, by BioRender.com (2020). Retrieved from https://app.biorender.com/biorender-templates (30 November 2021).

After fusion of the viral envelope with the cell membrane, the viral NC penetrates into the cytoplasm, where viral RNA transcription and replication occur (Figure 2). The viral RNA-dependent RNA polymerase (RdRp) L, associated with its cofactor P, is responsible for both activities [5,53]. Transcription of RSV also requires the viral protein M2-1, which acts as an anti-terminator/elongation factor [54,55], whereas MPV M2-1 is not essential for virus replication in cell culture [56]. During transcription, the RdRp has all the activities to transcribe, cap, and poly-adenylate mRNAs. Amplification of the viral genome by the RdRp necessitates the synthesis of an antigenome, which is also encapsidated by N. At the final stage of the viral cycle, NCs assemble with the other structural viral proteins at the cell surface to generate new virions (Figure 2).

More specifically, viral replication and transcription take place in cytoplasmic inclusions bodies (IBs), where all the proteins required for the activities of the RdRp concentrate [57] (Figure 2). These structures, also observed for others *Mononegavirales*, are membrane-less organelles that present liquid-like properties [58,59], and expression of N and P was shown to be sufficient to induce the formation of pseudo-IBs [60,61]. These IBs contain dynamic sub-compartments called IBAGs (IB-associated granules), where viral mRNA and the transcription factor M2-1 specifically accumulate [57] (Figure 2). It is noteworthy that hRSV proteins NS2 and M were also shown to localize to IBs [62,63,64]. Furthermore, different cellular proteins such as HSP70, actin, actin-associated proteins, translation initiation factors PABP, and eIF4G, as well as the phosphatase PP1, were shown to be recruited to IBs [57,65,66]. In particular, N was shown to interact with proteins involved in innate immune pathways such as MAVS, MDA5, and more recently, the subunit p65 of NF-κB, leading to their sequestration into IBs [67,68]. Thus, there is accumulating evidence that IBs are complex organelles that play a central role in the viral cycle, not only for viral RNA synthesis but also as platforms for the traffic of NCs from IBs to the plasma membrane and for assembly, as well as in the regulation of cellular innate immune responses to infection.

## 5. The Replication/Transcription Machinery of Pneumoviruses

**Figure 3:**
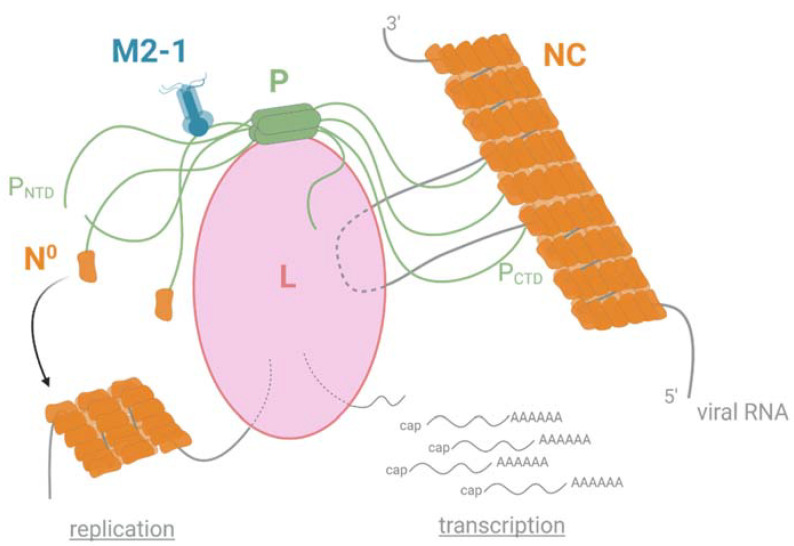
Schematic representation of the polymerase functioning of pneumoviruses. The polymerase L is responsible for both viral replication and transcription. The P protein plays a role in the hub by interacting with L and NC through its C-terminal P_CTD_ domain and with M2-1 and the monomeric and RNA-free N (N^0^) through its N-terminal P_NTD_ domain. Created with BioRender.com (30 November 2021).

The RdRp functioning depends on different PPIs, with the phosphoprotein P acting as a hub to recruit many partners, and more particularly by interacting with NC, L, M2-1, and the neosynthesized N (N^0^) (Figure 3).

The last decades were marked by the accumulation of structural and functional information on the pneumoviral RdRp. The main achievement was the recent determination of the 3D structure, although partial, of the L-P complexes of hRSV and hMPV by cryo-electron microscopy [69,70]. These structures revealed strong structural conservation between these two complexes, with a particular mode of P binding to L (see Section 6.1). They allowed establishing a model for the spatial functioning of L [70]. The structure and activities of the L protein will not be discussed extensively here. Briefly, the RdRp recognizes and uses the viral RNA genome as a template exclusively when it is encapsidated by N inside a flexible helical NC (Figure 3). This recognition is mediated by P, which is essential for loading the L polymerase onto the NC template and for keeping it bound to its template in a dynamic fashion during RNA synthesis.

The L protein embeds all enzymatic activities required for replication and transcription. The L protein contains an RdRp domain followed by a polyribonucleotidyl-transferase domain (PRNTase or capping domain) and a methyltransferase (MTase) domain. During transcription, the L protein scans the viral RNA, and mRNA synthesis begins at a conserved gene start sequence (GS). When the RNA is about 30 nucleotides long, a GMP moiety, covalently linked to the PRNTase domain of L, is transferred to the 5′ end of the nascent viral RNA, forming a cap structure (GpppG-RNA). The cap is subsequently methylated on its 2′ O and N7 position (N7GpppGm-RNA) by the MTase activity of L [71]. It is noteworthy that, although the mechanism still remains poorly understood, efficient hRSV transcription requires the recruitment of the M2-1 protein by P [54,55]. During replication, the RdRp synthetises antigenomes and genomes that are concomitantly encapsidated by the N protein. The assembly of new functional viral genomes requires a continuous supply of unassembled N molecules (N^0^). The P protein is an essential co-factor in this process by forming an N^0^-P complex to maintain N in a competent form for the encapsidation of new viral genomes (Figure 3).

## 6. Insights into P and N Protein Structures

The P and N proteins are the two main actors of the polymerase complex. Besides their direct role in viral RNA synthesis, they were shown to be the scaffold proteins responsible for IB morphogenesis [61,72]. This architectural role of P and N for IB formation was recently shown to depend on a liquid-liquid phase separation (LLPS) mechanism, requiring N-P interaction [73]. The LLPS mechanism is now well characterized. It is initiated by scaffold molecules that form condensates through the establishment of a network of interactions, more frequently, proteins and RNA. The archetype of protein architecture sustaining the formation of the LLPS relies on proteins with intrinsically disordered regions (IDRs) presenting multiple interacting motifs of low affinity [74,75,76] and RNA-interacting domains. The pneumoviruses P proteins, which present different IDRs and interact with NC, appear to be the pivotal element for IB morphogenesis [73].

### 6.1. The Modular Structure of the Phosphoprotein P

**Figure 4:**
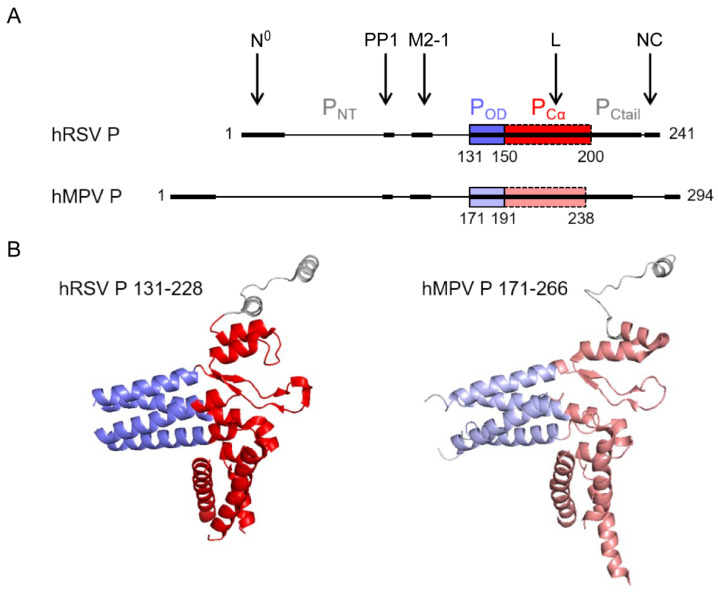
Structure of Pneumovirus P proteins. (**A**) Domain architecture of hRSV and hMPV P proteins, with a fully disordered N-terminal domain, P_NTD_, a short tetrameric coiled-coil oligomerization domain, P_OD_ (blue), and a C-terminal domain, P_CTD_, consisting of a domain with a high α-helical propensity, P_Cα_ (red), and a highly disordered C-terminal tail, P_Ctail_. The interaction regions of hRSV P with RdRp, or associated proteins like PP1, are indicated by arrows and bold lines. The corresponding regions in hMPV are also in bold lines. (**B**) High-resolution cryo-EM structures of the tetrameric L-associated hRSV and hMPV P proteins. Only the P_OD_ and P_Cα_ domains are observed in the L-P complex structures. Neither P_NTD_ nor P_Ctail_, except for a single protomer, could be observed due to high disorder. Created with Pymol (https://pymol.org, 30 November 2021).

Pneumoviral P proteins play a central role during the virus cycle, their high plasticity allowing the establishment of transient and complex interactions with various partners. Both hRSV and hMPV P proteins (of 241 and 294 residues, respectively) form parallel tetramers with a central oligomerization domain (P_OD_) consisting of a helical coiled-coil core, flanked by two intrinsically disordered regions (P_NTD_ and P_CTD_) (Figure 4A) [77,78,79,80,81]. Sequence alignment of the hRSV and hMPV phosphoproteins indicates a sequence identity and similarity of 28% and 38%, respectively, as calculated with the Sequence Manipulation Suite using an alignment made on the T-coffee server [82]. P_OD_ displays very high conservation with 65% identity and 80% similarity between hRSV and hMPV. P_Cα_, a subdomain of P_CTD_ with a high helical propensity, has 41% identity and 52% similarity. The P_NTD_ domain is longer in hMPV P than in hRSV P, but the N-terminus and the region proximal to the oligomerization domain also present conserved motifs that are likely molecular recognition elements (Figure 4A).

NMR proved to be a well-suited tool to obtain structural data of hRSV P alone in solution, and of its intrinsically disordered domains, in particular. This revealed that although these domains were not stably folded, several regions of P_NTD_ and P_CTD_ presented a propensity to form transient α-helices, likely to be involved in various PPIs [83,84,85]. These results were confirmed by interaction studies between P and N or M2-1 [65,83,86], but also by resolution of the 3D structures of P fragments in the complex with N^0^ [87], M2-1 [88], and L [69,70]. Whereas the interactions with N^0^ and M2-1 were shown to involve short linear motifs of the P_NTD_ domain that fold into helices upon binding, the recent structures of L-P complexes revealed that both P_OD_ and P_CTD_, the latter mostly through P_Cα_, interacts with L [69]. The location of these binding sites is indicated in Figure 4A. Interestingly, each P_CTD_ in the P tetramer was shown to adopt a specific and different conformation in contact with L (Figure 4B). However, the conformation of the L-bound hRSV and hMPV P_CTD_ tetramers is strikingly similar: structural alignment yields an RMSD of 1.224 Å. Of note, a recent study revealed that the hRSV P-M interaction involves both P_NTD_ and P_OD_ [64]. In contrast to these extended binding regions, the linear sequence corresponding to the last C-terminal residues of P was shown to be sufficient for binding to NC [89,90,91].

It is noteworthy that major and minor sites of phosphorylation were identified on hRSV P, with two main clusters of phosphorylated serines S116/S117/S119 and S232/S237 [92,93]. The phosphorylation status of P was shown to depend on cellular casein kinase II [94] and phosphatases PP1 and PP2A [93]. Although the role of these post-translational modifications during replication and transcription remains unclear [95,96,97], phosphorylation of hRSV P was shown to regulate its interaction with N and M2-1 [65,93,96,98]. hRSV P protein was also shown to recruit the phosphatase PP1 to IBs [65]. This interaction involves an RVxF-like motif of P located nearby and upstream of the M2-1 binding region (Figure 4A), which is conserved in hMPV P. Through its interaction with hRSV P, PP1 is involved in M2-1 dephosphorylation required for the efficient functioning of M2-1 and viral transcription. Therefore, phosphorylation is critical for the regulation of PPIs within the polymerase complex and efficient functioning.

### 6.2. Structure of Nucleoproteins

**Figure 5:**
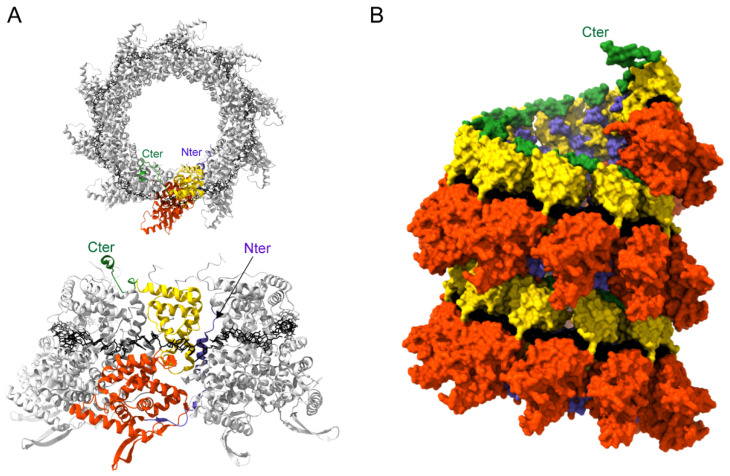
Structure of Pneumovirus N proteins. (**A**) Top (upper panel) and side (lower panel) views of hRSV N-RNA rings composed of 10 N proteins bound to RNA, purified from *E. coli* (PDB: 2WJ8 and 5FVC, respectively). N monomers are represented in ribbons; one N subunit is colored with the N_NTD_ in orange, N_CTD_ in yellow, and the N- and C-terminal parts in blue and green, respectively. The RNA is represented with the bases in black. N- and C-terminal extremities are indicated. (**B**) Left-handed N-RNA helix model (PDB: 4BKK). N monomers and RNA (black) atoms are shown as surfaces. One N subunit is colored with the N_NTD_ in orange, N_CTD_ in yellow, and the N- and C-terminal parts in blue and green, respectively. The C-terminal extremity of the N monomer at the top of the helix model is annotated. Created with UCSF ChimeraX [100].

The N protein, which is responsible for genome and antigenome encapsidation, is composed of 391 and 394 residues for hRSV and hMPV, respectively. This protein has a high binding affinity for RNA coupled with a strong tendency to oligomerize. The 3D crystal structure of the hRSV N expressed in *E. coli* and purified as annular ribonucleoprotein complexes composed of 10 N proteins bound to RNA (N-RNA rings) was first obtained in 2009 (Figure 5A) [99]. More recently, the structure of oligomeric N of hMPV, also purified as N-RNA rings, was also obtained [87]. These structures revealed strong structural conservation: N proteins have N- and C-terminal globular domains (N_NTD_ and N_CTD_, respectively) separated by a hinge region that forms the RNA-binding groove. Two flexible arms located at the N- and C-terminus of the protein bind to adjacent N protomers and rigidify the structure (Figure 5A).

The RNA wraps around the N protein ring in a basic groove, with seven nucleotides contacting each N monomer. The RNA is constrained and twisted by the N proteins, alternating rows of four and three stacked bases that are exposed and buried within the protein groove, respectively. Surprisingly, N-RNA rings, which were considered artifacts of production/purification, were recently found together with NCs in viral particles [101]. This raises the question of the potential role of these oligomers during the viral cycle. Electron microscopy analysis of hRSV NCs expressed in insect cells as well as cryotomography performed on viral particles revealed that these are left-handed helices [102,103]. Although the resolution of the helix was low, an atomic model of a left-handed RSV NC was generated (Figure 5B). These data allowed us to gain information on the interactions between N protomers of successive helix turns, and more importantly, to reveal that the 3′ end of the RSV genome is located at the pointed end of the NC. The structure of NC at high resolution still remains to be established to gain information on the mechanism sustaining the encapsidation of the viral genome and those required to allow genome accessibility to the polymerase.

Finally, during viral replication, the neo-synthesized N is maintained monomeric and RNA-free (N^0^) by P, which acts as a chaperone (see Section 7.2). Compared to the oligomeric form, N^0^ is characterized by a weak rotation of the N_NTD_ relative to the N_CTD_ and by the interaction of the N C-arm with the RNA groove, thereby preventing RNA binding.

## 7. N-P Interactions as Targets for New Antiviral Approaches

As previously mentioned, there is still no vaccine nor efficient antivirals against hRSV and hMPV. Most of the molecules under clinical trials target the fusion protein or the enzymatic activities of the L polymerase. However, the emergence of escape mutants upon treatments suggests that the combination of antivirals would be a necessity to prevent/limit the emergence of resistant viruses. The activity of the RdRp depends on many regulated PPIs. Among those, N-P interactions that are highly specific, of low affinity, and have no counterpart in cells, represent alternative targets for the development of new antivirals against hRSV and hMPV. Of interest, the structural data obtained for these PPIs now allow the rational design of inhibitors.

### 7.1. Interaction of P with NCs and Inhibition

**Figure 6:**
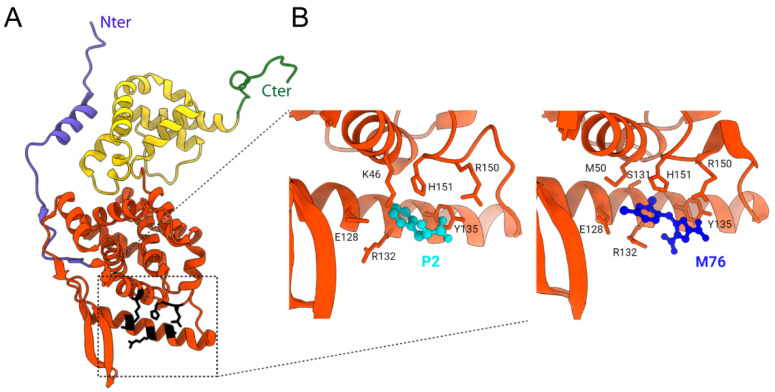
X-ray structure of hRSV N protein protomer and of N_NTD_ in complex with P2 peptide and M76 compound. (**A**) An hRSV N protomer taken from the X-ray structure of N-RNA rings (PDB 2WJ8) is represented in ribbons, with the N_NTD_ in orange, N_CTD_ in yellow, and the N- and C-terminal arms in blue and green, respectively. The residues of N critical for the interaction with P are shown with lateral chains in black. N- and C-terminal extremities are indicated. (**B**) Zoom into the P2-binding site on N_NTD_ (left panel, P2 in cyan) (PDB: 4UCA) and of the M76 compound binding site (right panel, M76 in deep blue) (PDB: 4UCC). The residues of N involved in the interaction with P2 or M76 are indicated. Created with UCSF Chimera [104].

By binding to N, P mediates the attachment of the L protein to the NCs. This interaction was well characterized for hRSV, and was recently shown to be essential for IBs’ biogenesis [73]. Using recombinant proteins, it was shown that the nine C-terminal residues of hRSV P are necessary and sufficient for binding to N-RNA rings [89]. More specifically, the C-terminal acidic and hydrophobic residues of P were shown to be critical for this interaction. Using a rational structure-based approach, the domain of hRSV N involved in P_CTD_ binding was then identified as N_NTD_ [90]. NMR interaction experiments showed that the last 10 C-terminal residues of P were involved in binding to N-RNA rings and N_NTD_, forming fuzzy complexes [83,91]. Residues of N critical for the interaction with P were found in a well-defined pocket composed of hydrophobic residues surrounded by positively charged residues [90]. These results were confirmed by the crystal structure of the N_NTD_ domain in the complex with the last two residues of P (P2 peptide in Figure 6) and highlighted the pivotal role of the P C-terminal residue Phe^241^, deeply buried in the N_NTD_ pocket [91]. It is noteworthy that this N pocket is accessible at the surface of the modelized helical nucleocapsid [102].

The low affinity of this N-P interaction coupled with the structural characterization of the P binding site on N suggested that small molecules could bind to the pocket of N to impair the P interaction. In silico screening of small compounds was thus performed to identify molecules that could insert into this P binding site. The M76 compound was able to compete in vitro with P for binding to N_NTD_ despite its low affinity (µM range). The crystallographic structure of the N_NTD_-M76 complex revealed that it displayed an optimal charge and shape for N binding (Figure 6B) [91]. However, M76 did not present any antiviral activity on infected cell cultures due to two acidic moieties preventing cell membrane passage. Further chemical modifications of M76 led to the electrically neutral pH-sensitive prodrug diAM-M76, which was internalized in cells. Although these modifications afforded a molecule with antiviral activity, diAM-M76 displayed a limited activity and important cytotoxicity [91]. Hence, although this approach validated the interest of targeting the P binding pocket of N, further chemical optimization of this compound is required to improve its affinity for N, as well as their cellular delivery and safety.

Other studies confirmed that this N-P interaction represents a potential target to develop antivirals against hRSV. Flavonoids such as hesperetin (Hst) or quercetin were initially shown to inhibit hRSV replication in vitro [105]. By coupling experimental and computational approaches, it was recently shown that Hst interacts with N, the aromatic ring of Hst being buried inside the hydrophobic N_NTD_ pocket involved in P binding [106], similarly to M76. High-throughput screening of a tailored library also led to the identification of the compound RSV-604 as a specific inhibitor of hRSV replication [107,108]. Further characterization of the mode of action of RSV-604 revealed that this benzodiazepine blocks both de novo synthesis of viral RNA and viral infectivity (assembly and release of virions) [109]. However, treatment of hRSV-infected cells with RSV-604 resulted in the emergence of resistant escape mutants, mutations being located in a region of N_NTD_ close to the P binding site. Based on these results, the compound EDP-938 has been developed [110]. Compared to RSV-604, EDP-938 displayed improved antiviral activity against hRSV, validated in non-human primates, but escape mutations also occurred on N. As mutations involved in the emergence of resistance concerned residues located close to the P binding site, it is tempting to speculate that these molecules could interfere with the N-P interaction. However, no structural nor experimental data supporting this hypothesis have been reported yet. Finally, a recent study showed that overexpression of P_CTD_ in cells inhibits hRSV replication, suggesting that a peptidomimetic approach could be developed to block the P-NC interaction [111].

Until recently, no information was available for the hMPV N-P interaction. Based on the strong structural and functional homologies between hMPV and hRSV N and P proteins, it was expected that N-P interactions could be partially conserved. Using site-directed mutagenesis approaches, coupled with in vitro and in cellula interaction assays, the last six C-terminal residues of hMPV P protein were shown to be necessary and sufficient for binding to N-RNA rings. Residues of P involved in this interaction, including the last C-terminal residue M^294^, are mostly hydrophobic [112]. Similar to hRSV, residues of N involved in the interaction were found at the surface of N_NTD_, and residues critical for P binding form a pocket mainly composed of hydrophobic residues, surrounded by charged residues [112]. Based on these results, molecular modeling was used to establish a model of binding between the last three C-terminal residues of P and N_NTD_. Altogether, these data confirmed the strong structural homologies between hMPV and hRSV P-N complexes but also highlighted some specificity. Therefore, whereas the hMPV P binding pocket at the N_NTD_ surface could also become a target for the rational design of hMPV antivirals, the design of dual inhibitors that could block both hMPV and hRSV N-P interaction is less likely.

### 7.2. The Pneumovirus N^0^-P Complex

The encapsidation process of neosynthesized genome and antigenome by N^0^ is an essential step of the viral cycle, the details of which are not yet fully understood. One main issue to gain information on the mechanism involved in the switch from N^0^ to N-RNA forms was to isolate and characterize a recombinant monomeric N^0^. Due to the N protein displaying a strong tendency to interact with RNA and to oligomerize, the purification of recombinant N^0^ has long been challenging. Different strategies were developed to prevent both RNA interaction and oligomerization of N. Initial approaches consisted in truncation of the N-terminal arm of N to impair N oligomerization [113,114] or substitution of N residues involved in RNA binding [115]. Using co-expression of recombinant mutated monomeric hRSV N protein with P fragments, the 29 N-terminal residues of P were shown to be sufficient to interact with N^0^ [114]. These results confirmed the predictions previously made based on sequence homologies between *Mononegavirales* [116], and the results obtained for its close homolog, the bRSV, showing an interaction between the N-terminus of P and N [117,118]. Moreover, the periodicity of the residues of hRSV P critical for N^0^ binding suggested that the stretch spanning residues 11–28 of P could adopt an α-helical conformation upon binding to N [115].

**Figure 7:**
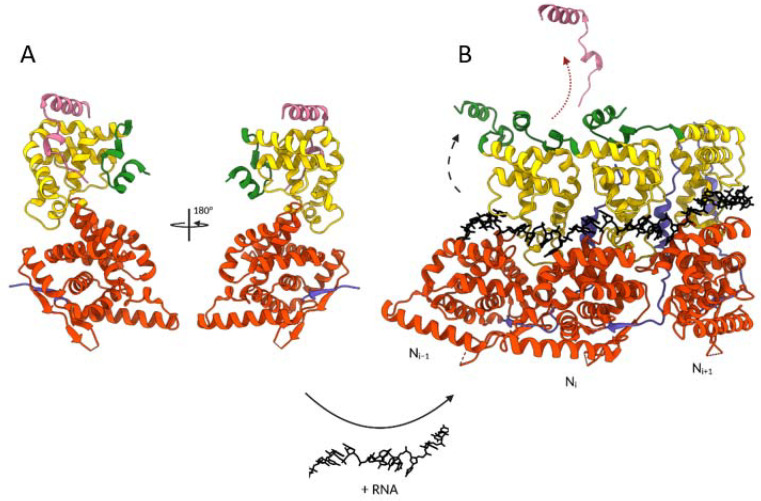
Structural changes involved in the transition from hMPV N^0^-P to oligomeric N-RNA forms. The transition requires the presence of RNA, as indicated at the bottom of the figure. (**A**) Crystal structure of hMPV N^0^- P_1-28_ peptide complex (PDB: 5FVD). (**B**) Representation of a trimer of N in cartoon representation bound to RNA shown in black sticks (PDB: 5FVC). The black and red arrows symbolize the movement of the N C-arm and the release of P_1–28_ peptide, respectively, required for N oligomerization. One N subunit is colored with the N_NTD_ in orange, N_CTD_ in yellow, and the N- and C-terminal parts in blue and green, respectively. The P_1-28_ peptide is in pink. The N- and C-terminal extremities of N and the P peptide are indicated. Created with BioRender.com and UCSF ChimeraX [100].

A major advance was made with the purification of a recombinant chimeric protein corresponding to full-length N fused with the N-terminal domain of P (first 40 residues), which allowed the resolution of the X-ray crystal structure of the hMPV N^0^-P complex (Figure 7) [87]. This structure revealed that the P peptide binds to a hydrophobic surface on N_CTD_. More specifically, residues 12–28 of P form an α-helix that lies atop N, and the N-terminal residues of P wrap along the N_CTD._ Interestingly, the P peptide binding site on N overlaps with the binding sites of both the N- and C-arms of the N_−i_ and N_+i_ protomers in the oligomeric form, preventing N self-oligomerization. This structure also revealed that the N^0^ form is characterized by a rotation of N_NTD_ relatively to N_CTD_, compared to the oligomeric form. Finally, the C-terminal arm of N was shown to fold into the positively charged RNA groove, blocking the binding of RNA.

Although the structure of the hRSV N^0^-P complex has not been solved yet, biochemical and biophysical studies confirmed the strong structural homology with hMPV. First, NMR characterization of hRSV P revealed that residues 12–24 present α-helical propensity [83]. Co-purification of hRSV N protein deleted of the N-arm (N_Δ30_) with 40 residues long peptide of P (P40) determined that the P binding site is located on N_CTD_ and that the C-arm of N is critical to prevent RNA binding [114]. Based on the biochemical and biophysical characterization of this N_Δ30_-P40 complex, the strong homologies between hRSV and hMPV N and P, and in particular, the strong conservation of key residues involved in the N^0^-P interaction, a structural model of the hRSV N^0^-P complex was proposed [114]. In contrast to N^0^-P complexes of other *Mononegavirales*, such as VSV, Nipah, Ebola, and Marburg viruses or parainfluenza 5 (PIV5), for which the binding of P was shown to be sufficient to maintain N^0^ [119,120,121,122,123,124,125], these observations demonstrated that the stability of pneumoviruses N^0^-P complexes requires a double lock system depending on P binding and on conformational changes of N (Figure 7).

Besides the identification of the hRSV P region involved in N^0^ binding, it was shown that the overexpression of a peptide with the corresponding sequence in cells could block the polymerase activity, giving a proof of concept that targeting the N^0^-P complex might be a way to develop novel antivirals against hRSV [114]. Accumulating data confirmed that the region encompassing residues 12–24 of P has a propensity to adopt an α-helical conformation that is stabilized upon binding [63,114,115]. Based on this observation, a strategy aiming at specifically targeting the N^0^-P interaction using dominant negative peptide inhibitors that mimic the N-terminus of P was recently developed [126]. These inhibitors were synthesized using the stapled peptide technology that constrains short peptides into α-helical conformation [127,128,129]. The presence of staples was shown to increase the potency, proteolytic stability, and cellular permeation of peptides. Of note, such a strategy was used to design peptide inhibitors of the hRSV F protein involved in the virus entry [130,131]. Screening of stapled peptides derived from the N-terminal sequence of P allowed identification of peptides derived from residues 7–30 of P, presenting an antiviral activity in cell culture, with an EC_50_ of approximately 10 µM [126]. Despite limited antiviral activity in cell cultures, the lead peptide was shown to reduce hRSV infection in vivo in a mouse model [126]. Because the N^0^-P interaction is mediated by a large interaction surface and involves a short α-helix, the stapled peptide approach is of greatest interest compared to small molecules. It is noteworthy that, given the strong structural homologies between hRSV and hMPV N^0^-P complexes, identification of the best combination of peptide length and stapling for hRSV would facilitate the design of peptides specific to hMPV.

## 8. Conclusions

The recent advances in the structure of the viral proteins associated with the L polymerase of pneumoviruses pave the way for the development of new antiviral strategies. Of particular interest, the PPIs required for the polymerase functioning represent potential targets for the design of new classes of antivirals. Indeed, these viral interactions are highly specific and have no cellular counterparts, suggesting that inhibitors should have limited off-target activity. Furthermore, because these interactions are transient and of low affinity, molecules of higher affinity should efficiently compete with the native mimicked sequence. Among these PPIs, the two modes of N-P interactions, which are now well characterized and relatively conserved between hRSV and hMPV, can be targeted using rational structure-based approaches. As described here, the design of potent inhibitors will depend on the nature of the PPIs: whereas small molecules seem promising to target the P binding site on oligomeric N and N-RNA complexes, such as the helical NCs, small peptides seem more adapted to inhibit the N^0^-P interaction. Similar approaches could be used to target L-P or M2-1-P interactions. It is noteworthy that these antiviral strategies could be applied to other *Mononegavirales*. Given the emergence of resistant escape viruses upon treatment with both anti-F and anti-L inhibitors, combinations of molecules directed against different viral targets may be required for efficient and long-term treatment.

## Figures and Tables

**Table 1:** Phylogeny of *Pneumoviridae*.

Family	Genus	Viruses
*Pneumoviridae*	*Metapneumovirus*	Human metapneumovirus (hMPV) Avian metapneumovirus (aMPV)
	Orthopneumovirus *	Human respiratory syncytial virus (hRSV) Bovine respiratory syncytial virus (bRSV) Pneumonia virus of mice (PVM)

* Unclassified viruses: Ovine respiratory syncytial virus (ORV), Canine pneumovirus (CPV), Swine orthpneumovirus (SOV).

## Data Availability

Not applicable.
